# Previously diagnosed multiple primary malignancies in patients with breast carcinoma in Western Sweden between 2007 and 2018

**DOI:** 10.1007/s10549-020-05822-z

**Published:** 2020-08-01

**Authors:** Jenny Nyqvist, Toshima Z. Parris, Khalil Helou, Elisabeth Kenne Sarenmalm, Zakaria Einbeigi, Per Karlsson, Salmir Nasic, Anikó Kovács

**Affiliations:** 1 Department of Surgery, Skaraborgs Hospital, Lidköping, Sweden; 2 Department of Oncology, Institute of Clinical Sciences, Sahlgrenska Cancer Center, Sahlgrenska Academy at University of Gothenburg, Gothenburg, Sweden; 3 Research and Development Centre, Skaraborgs Hospital, Skövde, Sweden; 4 Department of Oncology, Southern Älvsborg Hospital, Borås, Sweden; 5 Department of Oncology, Sahlgrenska University Hospital, Gothenburg, Sweden; 6 Department of Clinical Pathology, Sahlgrenska University Hospital, Gothenburg, Sweden; 7 Sahlgrenska Academy at University of Gothenburg, Gothenburg, Sweden

**Keywords:** Multiple primary metachronous and synchronous malignancies, Breast carcinoma, Gynecological malignancy, Malignant melanoma, Gastrointestinal malignancy

## Abstract

**Purpose:**

Multiple primary malignancies (MPMs) caused by breast cancer treatment are well described, but only few studies to date describe which other previous primary malignancies (OPPMs) occur before breast cancer. The purpose of the present study was to evaluate the prevalence of OPPMs in patients with breast cancer between 2007 and 2018 in Western Sweden.

**Methods:**

Patient selection was performed using both pathology reports at Sahlgrenska University Hospital (Sweden) and the Swedish Cancer Registry. All newly diagnosed breast cancer patients were screened for presence of OPPM.

**Results:**

In total, 8031 breast cancer patients were diagnosed at Sahlgrenska University Hospital between 2007 and 2018. The prevalence of breast cancer patients with OPPMs (*n* = 414) increased from on average 2.6% to 8.2% during this 12-year period and ranged from 17 to 59 patients annually. The most striking increase in prevalence was found among the gynecological tumors (endometrium and ovarian adenocarcinomas), malignant melanomas and gastrointestinal malignancies. These findings were validated using data of the Swedish Cancer Registry.

**Conclusions:**

The overall survival rates for cancer patients have improved tremendously during the past 40 years, in part due to individually tailored therapies and screening programs. Our study revealed an increasing trend of OPPMs in breast cancer patients.

## Introduction

During the past 40 years, overall survival rates have improved for cancer patients due to better prevention, earlier diagnosis with different screening programs and individually tailored therapies [1]. However, cancer patients are at a 10% elevated risk of developing additional primary malignancies in other organs [2, 3]. These so-called multiple primary malignancies (MPMs) are not metastases, but rather new primary tumors with differing histogenesis that can be easily verified by immunohistochemical examination. The phenomena of MPMs were described by Billroth et al*.* as early as 1889, followed by Warren and Gates in 1932 and Moertel in 1977 [4]. MPMs were defined as the coexistence of at least two unrelated primary malignancies in a single patient. Warren and Gates went a step further by stratifying MPMs into metachronous (> 6 months apart) and synchronous type (≤ 6 months in-between the primary malignancies).

An association between breast cancer and other primary malignancies is well established [5–12]. In 2008, Lee et al*.* described the risk of developing secondary malignancies in a cohort containing 53,783 Taiwanese women with breast cancer. Interestingly, young women (< 50 years of age) had a 43% elevated risk of developing other primary malignancies at any anatomical site, especially in bone, corpus uteri and ovary, skin (non-melanoma), thyroid, esophagus, kidney, lung, and leukemia or lymphoma. In contrast, older women (> 50 years of age) showed only excess risks of developing additional primary tumors in corpus uteri and ovary [13]. According to a population-based registry study compiled from 13 different cancer registries with 525,527 women with breast cancer from Europe, Canada, Australia, and Singapore, breast cancer survivors had a 25% increased risk of developing other primary cancers [14, 15].

MPMs may be caused by a variety of intrinsic, extrinsic, genetic, and therapeutic factors [16, 17]. Shared etiology may also play a pivotal role in the development of different malignancies due to the manifestation of common early genetic events at different time points, indicating a long latency period [18, 19]. Genome-wide association studies have identified 72 loci associated with breast cancer susceptibility, 17 of which are associated with MPM [20].

It is known that there are genetic syndromes associated with an increased susceptibility to breast cancer (e.g., Li-Fraumeni syndrome, Cowden syndrome, Lynch syndrome, Familial breast cancer: BROVCA1-4, Ataxia-telangiectasia, CDH1-associated breast cancer, PALB2-associated breast cancer, Peutz-Jeghers syndrome, Neurofibromatosis type 1), but it is possible that there are even more genetic syndromes to be detected [21, 22].

Studies have also shown that breast cancer patients have a 23–40% increased risk of developing a second primary malignancy after anti-hormone therapy (e.g., endometrium adenocarcinoma), chemotherapy (e.g., leukemia), and radiotherapy (e.g., sarcoma, esophagus and thyroid gland malignancy [23]. However, chemotherapy administered for the first breast cancer is also associated with a decreased risk of developing other primary malignancies, suggesting that chemotherapy might have a protective effect for subsequent/simultaneous undiscovered colorectal, lung, ovarian and head and neck cancer. Moreover, women with bilateral breast cancer have as much as 1.5 times higher risk of developing non-breast cancer-related malignancies [24].

Previous studies have suggested that breast cancer treatment could play a crucial role in the development of other primary malignancies as a late effect and consequence of the treatment [14, 15, 23]. However, only few studies have been published regarding the incidence of other previous primary malignancies (OPPMs) that occur before a breast cancer diagnosis. Though, there are earlier studies about MPM, but the focus was not specifically on subsequent second primary breast carcinoma [22, 25, 26]. Our hypothesis as breast cancer researchers was that other primary malignancies even occur and increasing before breast cancer diagnosis. Our first aim was to see what kind of malignancies has inclination to future breast cancer. One of the reasons for high lightning this phenomenon is based on our interest for breast cancer itself. Therefore, we designed the study in this way. Another aim was to predicate which prior malignancies mean risk for future breast cancer development. This could result in additional screening programs and genetic analysis for prevention of future breast cancer or earlier detection. It is also important to make differential diagnosis between primary tumors and metastases resulting in better clinical outcome in case of primary tumors.

We evaluated the incidence of OPPMs in two steps. First, 8031 patients diagnosed with breast cancer between 2007 and 2018 in Western Sweden using the data system of the Department of Clinical Pathology at Sahlgrenska University Hospital, Gothenburg. Second, we performed a population-based study using data from 5132 patients with breast cancer collected from The National Board of Health and Welfare.

## Methods

### Study participants and data collection

#### Data collection from the Sahlgrenska University Hospital Registry (2007–2018)

Patients were eligible for inclusion in the study if they were newly diagnosed with primary invasive breast cancer and had previous malignancies. In total, 8031 breast cancer patients diagnosed at Sahlgrenska University Hospital (Gothenburg, Sweden) between 2007 and 2018 were selected for inclusion in the study. Patient selection was performed using pathology reports in the Sympathy database (from 1970s to present) and written patient records from multidisciplinary cancer conferences (from 2007 to present) at the Department of Clinical Pathology (Sahlgrenska University Hospital). The Western Board of Cancer Registry (Regionala Cancer Centrum i Väst) verified the number of newly diagnosed breast cancer cases and the number of OPPMs reported before each breast cancer case. Access to the immunohistochemistry data was available for each patient and tumor. All malignant tumors of non-breast origin were verified immunohistochemically to exclude metastases.

#### Population-based data collection of the Swedish Cancer Registry (2007–2017)

To establish whether the increasing prevalence of OPPMs in the examined period (an increase from 2.6 to 8.2% between 2007 and 2018, mainly according to Pathology Department database Sympathy) was reliable in an epidemiological population-based perspective, data for breast cancer patients diagnosed between 2007 and 2017 with OPPMs was also retrieved from the Swedish Cancer Registry at the National Board of Health and Welfare (Socialstyrelsen) for four municipalities in the Gothenburg region (Gothenburg-, Härryda-, Mölndal- and Kungälv municipalities). A possible confounding factor is differences in the attachment area of the Sahlgrenska University Hospital during the years. Therefore, we had to specify which municipalities we wanted to involve when applying for statistical data from the National Registry.

The Swedish Cancer Registry started in 1958. Therefore, the follow-up time was defined as the time between the date of breast cancer diagnosis and the OPPM diagnosis from 1958, being 49 years the longest period before the onset of breast cancer diagnosis (according to The Cancer Register) to ensure the same length of time at risk. The coverage rate of the registry is almost 100%. Male patients and very unusual types of malignancies were excluded from the data collection from the Swedish Cancer Registry due to integrity issues. Metastases were excluded because our aim was to study primary malignancies. Benign tumors were excluded then we study malignant tumors. Common skin tumors (e.g., basal cell carcinoma and squamous cell carcinoma) were excluded then they often appear on elderly people.

With these data from the National Board of Health and Welfare, we could also avoid the risk of missing patients due to relocation and missing data about any previous malignancies. The ICD7 (International Statistical Classification of Diseases and Related Health Problems, WHO classification of diseases from 1952; ICD7 from 1958) and the histopathology diagnosis codes (SNOMED) were used to find patients and their malignancies in the Swedish Cancer Registry. We investigated which malignancies each patient was diagnosed with before their breast cancer and in what order the OPPMs appeared. The index (the year of breast cancer diagnosis) were those patients who were diagnosed with breast cancer between 2007 and 2017. Due to integrity issues, patients were divided into age categories at 10-year intervals (< 49, 50–59, 60–69, 70–79, 80+). Using this multistep procedure, we were able to evaluate the prevalence of OPPMs in breast cancer patients diagnosed from 2007 to 2017.

### Statistical analysis

Descriptive statistics was presented as frequencies and percentages for categorical variables and as mean and range for continuous variables. For group comparisons with respect to categorical variables, the Chi-square test was used. A possible change over time with respect to frequencies/percentages of patients with another primary malignancy was tested by “linear-by-linear” Chi-Square test and by logistic regression with MPM (yes or no) as outcome and year as explanatory variable. *p* value < 0.05 was considered as statistically significant if nothing else mentioned. The IBM SPSS v.25 statistical package was used for statistical analyses.

## Results

### The prevalence of OPPMs (mainly, malignant melanomas, gynecological and gastrointestinal cancers) in breast cancer patients significantly increased during 2007–2018

**Fig. 1:**
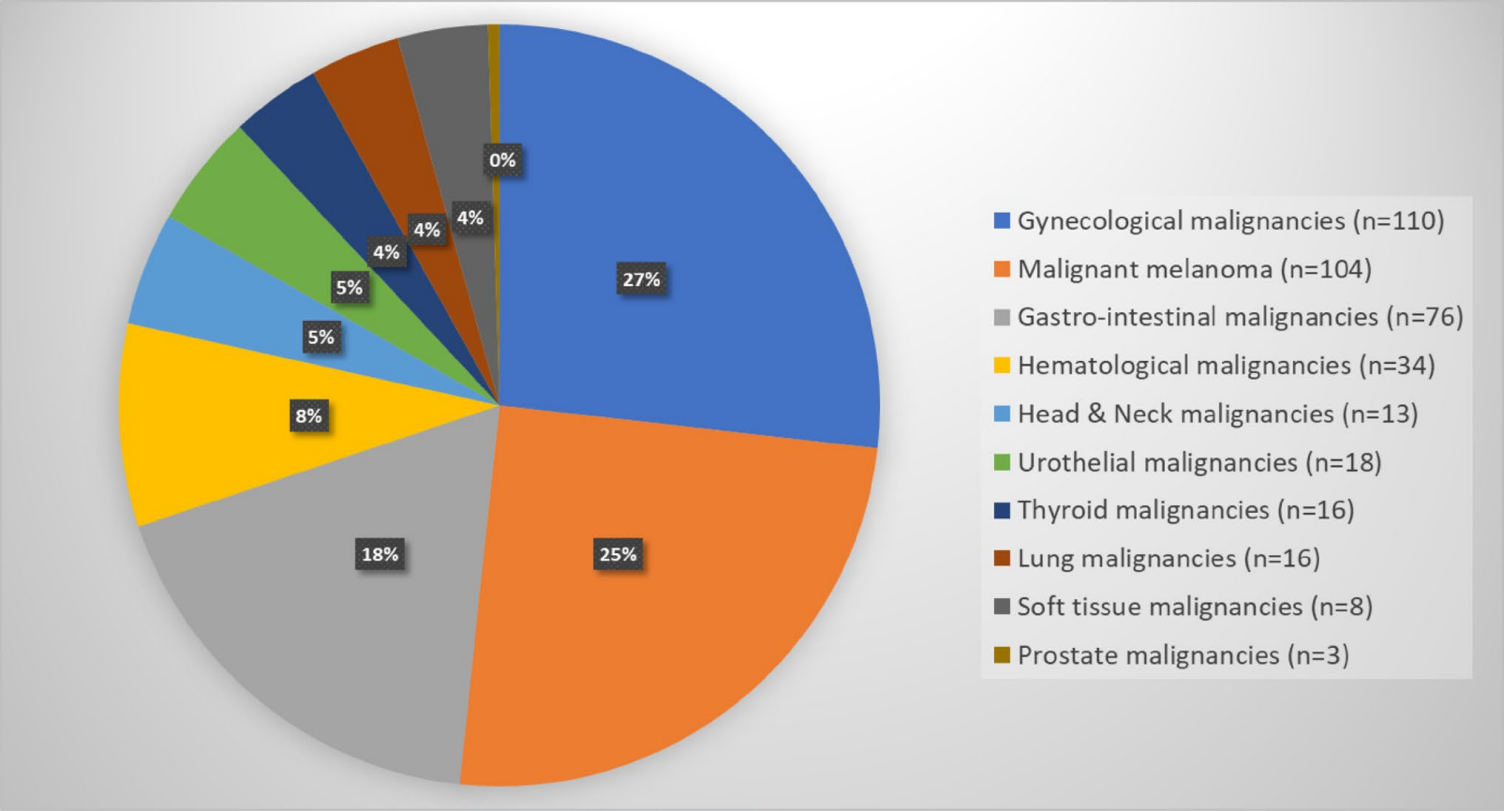
Distribution of MPMs in breast cancer patients according to pathology reports from Sahlgrenska University Hospital (2007–2018)

In total, 8031 patients were diagnosed with breast cancer between 2007 and 2018 at Sahlgrenska University Hospital, of which 414 patients had previously been diagnosed with OPPMs (Table 1). The average patient age was 68 (range 36–94 years of age). In 2007, 3.3% of the newly diagnosed breast cancer patients (n = 545) were found to have OPPMs, while this number had risen to 6.6% in 2018. However, the highest prevalence of patients with OPPMs was found to be 8.2% in 2016 and the lowest prevalence was 2.6% in 2008 (Table 1). The prevalence of OPPMs in breast cancer patients increased over time during this 12-year period (*p* < 0.001). Of the 414 patients with breast cancer and OPPMs, 18 patients had more than one additional OPPMs before their breast cancer diagnosis (16 patients had two OPPMs, one patient had three OPPMs, and one patient had four OPPMs). OPPMs comprised gynecological cancer (27%), malignant melanoma (25%), and gastrointestinal carcinoma (18%) were most prevalent, whereas thyroid cancer (4%), lung carcinoma (4%), and soft tissue malignancies (4%) were least common (Fig. 1).

**Table 1:** The distribution of patients with newly diagnosed breast cancer and previous MPMs according to pathology reports from Sahlgrenska University Hospital (2007–2018)

	No. of patients (%)	
	Patients with newly diagnosed breast cancer (2007–2018) (n = 8031)	Breast cancer patients with other previously diagnosed primary malignancies (2007–2018) (n = 414)
2007	545 (89% invasive, 11% in situ)	18 (3.3%)
2008	657 (86% invasive, 14% in situ)	17 (2.6%)
2009	521 (83% invasive, 17% in situ)	21 (4.0%)
2010	688 (84% invasive, 16% in situ)	32 (4.7%)
2011	678 (88% invasive, 12% in situ)	29 (4.3%)
2012	634 (88% invasive, 12% in situ)	29 (4.6%)
2013	731 (86% invasive, 14% in situ)	27 (3.7%)
2014	800 (85% invasive, 15% in situ)	54 (6.8%)
2015	745 (86% invasive, 14% in situ)	49 (6.6%)
2016	718 (87% invasive, 13% in situ)	59 (8.2%)
2017	725 (87% invasive, 13% in situ)	40 (5.5%)
2018	589 (83% invasive, 17% in situ)	39 (6.6%)

*p* value < 0.001 (*p* value for trend over time, tested by Poisson regression for counts and by linear regression with rates/fractions as outcome, *p* value < 0.001 in both cases)

Generally, the breast carcinomas of patients with OPPMs were frequently invasive ductal carcinomas (63.9%) and histological grade 2. On average, estrogen receptor (ER)-positivity, progesterone receptor (PgR)-positivity, Ki-67-positivity, and HER2 amplification was 84%, 55%, 26%, and 8.7%, respectively. A comparison between the breast carcinomas of patients diagnosed with and without OPPMs revealed no difference in ER-positivity (84% in patients with OPPMs versus 86% in patients only with breast cancer) but lower expression of PgR in patients with OPPMs (55% in patients with OPPMs versus 71.4% in patients only with breast cancer) and more frequent axillary lymph node metastasis (29.5%) in patients with OPPMs.

### The Swedish Cancer Registry validates the most common OPPMs in breast cancer patients

Evaluation of data from the Swedish Cancer Registry revealed that 5132 patients were diagnosed with breast cancer in the Gothenburg region (Gothenburg-, Kungälv-, Mölndal- and Härryda municipalities) during 2007–2017. In total, 4659 (90.8%) of the 5132 patients were only diagnosed with breast cancer and 473 patients (9.2%) had two or more primary malignancies (507 tumors; including breast cancer). Bilateral or multifocal breast cancer were counted as one malignancy because of the same histopathological origin. Interestingly, 44 patients had three or more primary malignancies including breast cancer and six patients had four or more primary malignancies including breast cancer.

**Fig. 2:**
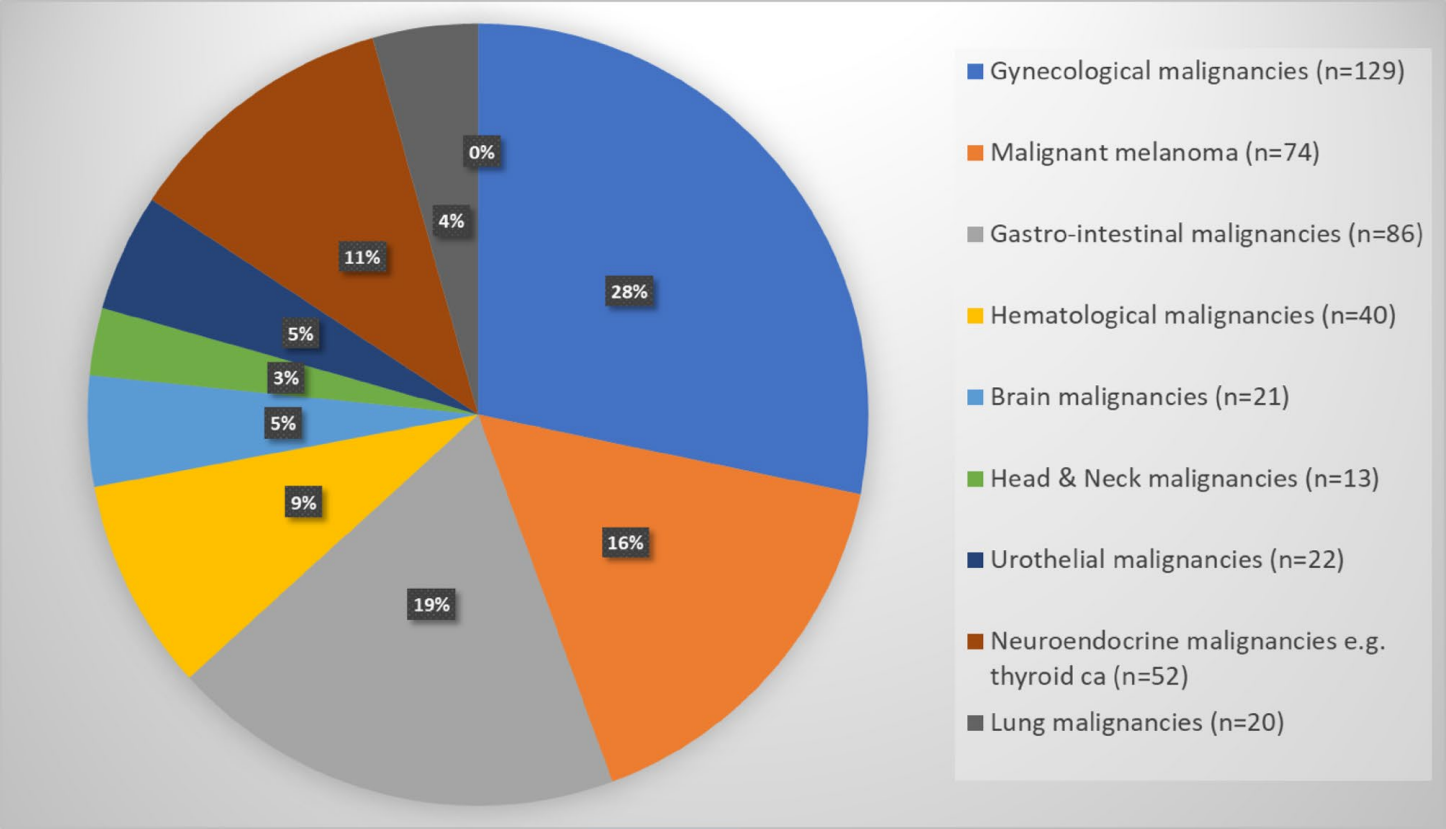
Distribution of MPMs in breast cancer patients according to the Swedish Cancer Registry (2007–2017)

In agreement with data from Sahlgrenska University Hospital, the number of OPPMs increased during 2007–2017 from 8.1 to 10.4%. Nevertheless, this increasing trend was not statistically significant with either “linear-by-linear” Chi-2 test or logistics regression (*p* value = 0.075; Table 2)*.* In addition, gynecological malignancies (n = 129, 28%), skin cancer (n = 116, 24.5%, including malignant melanoma n = 74, 16%), and gastrointestinal malignancies (n = 86, 19%) were found to be the most common primary malignancy in patients with OPPMs (except breast cancer), while the least common other malignancies including malignant soft tissue tumors (*n* = 5, 1%). Furthermore, 34/129 gynecological malignancies were ovarian cancer (7.2%) and 74/116 skin cancers were malignant melanoma (16%; Fig. 2).

**Table 2:** The distribution of patients with newly diagnosed breast cancer and previous MPMs according to the Swedish Cancer Registry (2007–2017)

	No. of patients (%)	
	Patients with newly diagnosed breast cancer (2007–2017) (*n* = 5132)	Breast cancer patients with other previously diagnosed primary malignancies (2007–2017) (*n* = 473)
2007	394	32 (8.1%)
2008	468	37 (7.9%)
2009	347	19 (5.5%)
2010	471	53 (11.3%)
2011	485	41 (8.5%)
2012	439	45 (10.3%)
2013	477	44 (9.2%)
2014	520	55 (10.6%)
2015	506	44 (8.7%)
2016	506	49 (9.7%)
2017	519	54 (10.4)

*p* value = 0.075 (*p* value for trend over time, tested by Poisson regression for counts and by linear regression with rates/fractions as outcome, *p* value = 0.075 in both cases)

## Discussion

Cancer incidence rates are steadily increasing and by 2030 over 21 million individuals are estimated to receive a cancer diagnosis, with an increasing incidence by 1.7% per year [1, 13, 23]. Due to longer life expectancies, earlier diagnosis and more individually tailored cancer treatments, incidence rates for MPMs are increasing. In addition to intrinsic-, extrinsic- and environmental factors, MPMs may also be caused by genetic alterations and therapy-related factors. In those patients with MPMs without detectable extrinsic factors and known genetic mutations or syndromes, it is unlikely that this would be a mere coincidence. To avoid the confounding factor of therapy-related malignancies, we focused on OPPMs diagnosed before the breast cancer diagnosis and subsequent treatment.

In agreement with previous studies regarding MPM in general (when a other primary malignancies can occur both before and after breast cancer), we show a statistically significant increase in incidence rates during 2007–2018 for breast cancer patients with OPPMs using pathology reports in hospital records [14, 15, 23, 27]. Although data from the Swedish Cancer Registry showed the same trend, it was not statistically significant possibly due to missing data from the Swedish Cancer Registry. This could be due to several factors connected with cohort selection, e.g., exclusion of men, limited selection of geographical population, and missing data. Use of registry data has been known to present some limitations, e.g., incomplete data, differences in population categorization and altering coding systems, and the human factor [28]. However, we do know that the Swedish Cancer Registry has a high factor of reliability. Though, among the statistical data from the National Registry we noticed that some tumors were named as tumors appearing directly as number two or number three. It could have happened that the first tumor was diagnosed before 1958, when the National Registry was established. Therefore, our two study cohorts (Sahlgrenska University Hospital and the Swedish Cancer Registry) cannot be compared. We are also aware that the pathology reports for the different tumors could differ due to subsequent development of analytical and immunohistochemical methods, as well as, biopsy techniques over time.

Furthermore, both data sources demonstrated that malignant melanoma, gynecological malignancies and gastrointestinal tumors most often preceded the breast cancer diagnosis. To the best of our knowledge, no similar studies have assessed the prevalence of OPPMs before a breast cancer diagnosis. However, several previous studies describe MPMs after a breast cancer diagnosis [14–16, 23, 27, 29–36]. Mellemkjaer et al*.* described that women had more than doubled risk of developing soft tissue sarcoma, as well as increased risk of developing thyroid cancer (60%), non-melanoma skin cancer (58%), endometrial cancer (52%), ovarian cancer (48%), stomach cancer (35%), melanoma (29%), kidney cancer (27%), lung cancer (24%), and colorectal cancer (22%) [15].

In summary, we show a steady increase in prevalence of OPPMs in breast carcinoma patients during 2007–2018 according to the pathology reports from Sahlgrenska University Hospital. However, the attempt to a validation through a population-based cohort with data from the Swedish Cancer Registry (2007–2017) could only show an increasing tendency without any significance.

Furthermore, the most common OPPMs included malignant melanoma, gynecological malignancies and gastrointestinal tumors. Increased cancer prevalence places great demands on society. These challenges must be met with better prevention, effective and well-organized cancer care, and investment in high-quality cancer research. Identifying the type of different coexisting primary malignancies may awaken clinical vigilance among oncologists, warranting new screening programs for cancer patients to detect certain second or third, etc., primary malignancies at an early stage. An interdisciplinary collaboration is needed to guarantee the best treatment outcome for these OPPM patients. If we can predict who will most likely suffer from OPPMs, it can be beneficial both socio-economically and personally. Therefore, not only treatment tailoring, but follow-up programs should be considered to prevent further malignancies at a late stage. Taken together, further studies are warranted to establish the role genetic alterations may have in the development of OPPMs in certain risk groups. That is the reason why we initiated a subsequent study to genetically analyze both the previous malignant tumor and the following breast cancer which is to be published.

## Data Availability

The datasets used and analyzed during the current study are available from the corresponding author on reasonable request.

## Abbreviations

ER: Estrogen receptor
HER2: Human epidermal growth factor receptor 2
MPM: Multiple primary malignancies
OPPM: Other previous primary malignancies
PgR: Progesterone receptor
